# Towards a brief definition of burnout syndrome by subtypes: Development of the "Burnout Clinical Subtypes Questionnaire" (BCSQ-12)

**DOI:** 10.1186/1477-7525-9-74

**Published:** 2011-09-20

**Authors:** Jesús Montero-Marín, Petros Skapinakis, Ricardo Araya, Margarita Gili, Javier García-Campayo

**Affiliations:** 1 Department of Psychiatry. University of Zaragoza. REDIAPP (Research Network on Preventative Activities and Health Promotion, RD06/0018/0017). Spain; 2 Faculty of Health and Sports. University of Zaragoza, Huesca. Spain; 3 Academic Unit of Psychiatry, School of Social and Community Medicine, University of Bristol, UK; 4 Department of Psychiatry, University of Ioannina School of Medicine, Ioannina, Greece; 5 Institut Universitari d'Investigació en Ciències de la Salut (IUNICS), University of Balearic Islands, REDIAPP (Research Network on Preventative Activities and Health Promotion, RD06/0018/0017). Spain

**Keywords:** burnout, subtypes, BCSQ-12, factorial validity, criterial validity

## Abstract

**Background:**

Burnout has traditionally been described by means of the dimensions of exhaustion, cynicism and lack of eficacy from the "Maslach Burnout Inventory-General Survey" (MBI-GS). The "Burnout Clinical Subtype Questionnaire" (BCSQ-12), comprising the dimensions of overload, lack of development and neglect, is proposed as a brief means of identifying the different ways this disorder is manifested. The aim of the study is to test the construct and criterial validity of the BCSQ-12.

**Method:**

A cross-sectional design was used on a multi-occupational sample of randomly selected university employees (n = 826). An exploratory factor analysis (EFA) was performed on half of the sample using the maximum likelihood (ML) method with varimax orthogonal rotation, while confirmatory factor analysis (CFA) was performed on the other half by means of the ML method. ROC curve analysis was preformed in order to assess the discriminatory capacity of BCSQ-12 when compared to MBI-GS. Cut-off points were proposed for the BCSQ-12 that optimized sensitivity and specificity. Multivariate binary logistic regression models were used to estimate effect size as an odds ratio (OR) adjusted for sociodemographic and occupational variables. Contrasts for sex and occupation were made using Mann-Whitney U and Kruskall-Wallis tests on the dimensions of both models.

**Results:**

EFA offered a solution containing 3 factors with eigenvalues > 1, explaining 73.22% of variance. CFA presented the following indices: χ^2 ^= 112.04 (p < 0.001), χ^2^/gl = 2.44, GFI = 0.958, AGFI = 0.929, RMSEA = 0.059, SRMR = 0.057, NFI = 0.958, NNFI = 0.963, IFI = 0.975, CFI = 0.974. The area under the ROC curve for 'overload' with respect to the 'exhaustion' was = 0.75 (95% CI = 0.71-0.79); it was = 0.80 (95% CI = 0.76-0.86) for 'lack of development' with respect to 'cynicism' and = 0.74 (95% CI = 0.70-0.78) for 'neglect' with respect to 'inefficacy'. The presence of 'overload' increased the likelihood of suffering from 'exhaustion' (OR = 5.25; 95% IC = 3.62-7.60); 'lack of development' increased the likelihood from 'cynicism' (OR = 6.77; 95% CI = 4.79-9.57); 'neglect' increased the likelihood from 'inefficacy' (OR = 5.21; 95% CI = 3.57-7.60). No differences were found with regard to sex, but there were differences depending on occupation.

**Conclusions:**

Our results support the validity of the definition of burnout proposed in the BSCQ-12 through the brief differentiation of clinical subtypes.

## Background

Burnout syndrome is considered a uniform condition with relatively consistent aetiology and symptoms resulting from prolonged exposure to chronic stressors in the workplace [1]. This syndrome tends to be given standard operationalization through the "Maslach Burnout Inventory General Survey" (MBI-GS) by means of the dimensions of 'exhaustion', 'cynicism' and professional 'inefficacy' [2]. 'Exhaustion' is the feeling of not being able to offer any more of oneself at an emotional level; 'cynicism' is contemplated as a distant attitude towards work; and 'inefficacy' is the feeling of not performing tasks adequately.

Clinical experience, however, shows that burnout is manifested in different ways that can be classified depending on the level of dedication with which individuals cope with work-related tasks [3,4]. The "frenetic" burnout sub-type is characterized by the investment of a large amount of time to work and is common in highly involved, ambitious and overloaded individuals. 'Involvement' is the investment of every effort required to overcome difficulties; 'ambition' is a great need to obtain important success and achievements at work; and 'overload' is risking one's own health and neglecting of one's own personal life in the pursuit of good results [4-7]. The "underchallenged" burnout subtype is influenced by the occupation type. It appears in indifferent and bored individuals who do not find personal development in their work. 'Indifference' is lack of concern, interest and enthusiasm in work-related tasks; 'boredom' is caused by the understanding of work as a mechanical and routine experience with little variation in activities; and 'lack of development' is the absence of personal growth experiences for individuals together with their desire for taking on other jobs where they can better develop their skills [4-7]. The "worn-out" burnout subtype is determined by the rigidity of the organizational structure of an individual's workplace and is characterized by a lack of control over results, lack of recognition for efforts and neglect of responsibilities. 'Lack of control' is the feeling of helplessness as a result of dealing with many situations that are beyond their control; 'lack of acknowledgement' is the belief that the organizations those individuals work for fail to take their efforts and dedication into account; and 'neglect' refers to individuals' disregard as a response to any difficulty [4-7].

This conceptualization of burnout, operationalized through the "Burnout Clinical Subtype Questionnaire" (BCSQ-36), is very useful for the specific evaluation of the syndrome and for the design of treatment strategies depending on the characteristics of each clinical case. This is practicable given that it provides a broader framework that exceeds the possibilities for evaluation and intervention implicit in the standard design of the MBI-GS, which is more directed towards a unified (although three-dimensional) definition of the syndrome [7,8].

The dimensions of 'overload', 'lack of development' and 'neglect', belonging to the subtypes of "frenetic", "underchallenged" and "worn-out", respectively, could construct a brief definition of burnout that is able to bring the typological perspective of the BCSQ-36 closer to the MBI-GS standard [8]. These dimensions have been proposed as a definition of burnout that could cover common ground between the typological and standard approaches, and have been selected as a result of a second order factor analysis, carried out between the dimensions of BCSQ-36 and MBI-GS taken together [1,2,4,7,8]. These dimensions showed good discriminant validity, which makes them very useful for the brief identification of clinical subtypes of burnout [8]. However, it is necessary to explore and confirm the structure of this new definition, in view of the fact that it groups the items of the original scale in a different way. It will also be necessary to analyse its criterion validity because this new design reduces the extent of the initial typological definition.

The main objectives of this study were to test the factorial structure of the differential design proposed by means of the dimensions of 'overload', 'lack of development' and 'neglect' through the BCSQ-12, and to estimate its discriminatory strength compared to the dimensions of 'exhaustion', 'cynicism' and 'inefficacy' of the MBI-GS standard. We also proposed to evaluate the internal consistency of the dimensions and possible differences caused by gender and occupation.

## Method

### Design and study population

A cross-sectional design was utilized by means of the self-report technique through an online questionnaire completed by selected subjects who had provided informed consent.

The study population was comprised of the entire workforce of the University of Zaragoza in employment in January 2008 (N = 5,493). The sample size was calculated with a 95% confidence interval and a margin of error of 3.5%. The prevalence of burnout was estimated at 18% [9], giving a result of 427 subjects. As the expected response rate in web-mail surveys is approximately 27% [10,11], and in order to perform both an exploratory and confirmatory factor analysis on the different groups, 3,200 employees were selected by stratified probability sampling with proportional allocation by occupation (58% teaching and research staff or 'TRS', 33% administration and service personnel or 'ASP' and 9% trainees or 'TRA').

The participants' total final sample (n_T _= 826) was divided randomly into two equal halves (n_1 _= 413 and n_2 _= 413). The size of the resulting sub-samples permitted the established margin of error to be maintained and exceeded the construct validity evaluation criterion, making it possible to perform the analysis on both groups with psychometric adjustment [12-15]. The sample size calculation, subject selection and sample division were performed with Epidat 3.1. software.

### Procedure

An e-mail was sent to the selected subjects explaining the aims of the research. This message contained a link to an online questionnaire and two access passwords that enabled the subjects to complete the questionnaire during the month of February 2008. The first page of the protocol again provided another explanation of the aims of the study, the participants to whom it was addressed, the voluntary nature of participation in it, possible benefits/risks entailed and the confidentiality of information given. All participants received an anonymous report with an explanation of their results. The project was approved by the regional Clinical Research Ethics Committee of Aragon.

### Measurements

#### Sociodemographic and Occupational factors

Subjects were first asked a set of questions dealing with socio-demographic and occupational characteristics including: age, sex, whether they were in a stable relationship ('yes' vs 'no'), level of education ('secondary or lower', 'university degree', 'doctorate'), occupation type ('TRS', 'ASP', 'TRA'), years of service (' < 4', '4-16', ' > 16'), type of employment contract ('permanent' vs 'part time') and whether they had taken sick leave in the previous year ('yes' vs 'no').

#### Burnout Clinical Subtype Questionnaire (BCSQ-12)

Following on, they were provided with the "Burnout Clinical Subtype Questionnaire" in its brief Spanish version, the BCSQ-12 (Additional file 1, Appendix 1: Spanish language version of BCSQ-12; Appendix 2: English language version of BCSQ-12). This questionnaire consists of 12 items equally distributed between the dimensions of 'overload' (e.g. "I overlook my own needs to fulfil work demands"), 'lack of development' (e.g. "My work doesn't offer me opportunities to develop my abilities") and 'neglect' (e.g. "When things at work don't turn out as well as they should, I stop trying"). Subjects had to indicate their degree of agreement with each of the statements presented according to a Likert-type scale with 7 response options, scored from 1 (totally disagree) to 7 (totally agree). The results were presented as scalar scores. Cronbach's α coefficient showed the internal consistency of these dimensions, with values of α≥0.85 in all cases in the present study.

#### Maslach Burnout Inventory General Survey (MBI-GS)

Subjects were also given the "Maslach Burnout Inventory-General Survey" (MBI-GS) [2] in its validated Spanish language version [16]. This adaptation consists of 15 items grouped into 'three dimensions: 'exhaustion' (e.g. "I feel emotionally drained from my work"), 'cynicism' (e.g. "I've become more callous towards people since I took this job") and 'efficacy' (e.g. "I deal very effectively with the problems of my work"). Responses were arranged (in a Likert = type scale with 7 response options, scored from 0 ('never') to 6 ('always'). Results are presented in scalar scores. All of the questionnaire dimensions acquired an internal consistency of α≥0.78 [16].

### Data analysis

A descriptive analysis of the participants' socio-demographic and occupational characteristics was conducted, using means and standard deviations for age and percentages for the other variables. Contrasts were made depending on the sub-sample to which participants belonged using Student's t-test for age and χ^2 ^for the rest.

An initial contrast was made of the validity of the BCSQ-12 construct by means of an exploratory factor analysis (EFA) over n_1_. The maximum likelihood (ML) extraction method was used with varimax orthogonal rotation to facilitate interpretation, enabling relatively unrelated dimensions to be obtained. We had previously verified that: the correlations matrix presented a large number of significant values; all variables presented a value of r > 0.30; the absolute values of the anti-image matrix were close to 0; the matrix determining factor was very low; the Kaiser-Meyer-Olkin (KMO) index was > 0.70; Barlett's test of sphericity was statistically significant; and the measures of sampling adequancy (MSA) were above 0.80 [13]. The number of components was decided using Kaiser's criterion, which requires eigenvalues > 1 [17], in addition to Cattel's scree test on the sedimentation graph [18]. The belonging factor was determined by means of the factor weight criterion *w *> 0.5 in only one of the factors [12] and the percentage of variance explained in each variable by means of h^2 ^communality values.

Confirmatory factor analysis (CFA) was performed over n_2 _in order to ensure the clear distinction between the factors. The covariance matrix was used for data entry as it enables robust analysis to be made of ordinal data when the latent variables present more than one indicator [19]. This analysis was carried out using the ML method. This method assumes a multivariate normality, although it is relatively insensitive to its non-observance [20,21]. Nevertheless, we ensured that Mardia's coefficient for kurtosis was < 70 [22], given that below this limit, the ML method provides consistent parameter estimates [23]. All components of the model were introduced as latent factors, taking the items of the BCSQ-12 as observable variables distributed according to the original proposal [7]. From an analytical perspective, factor saturations (λ) > 0.5 [24-26], the explained variance on each observable variable (*R^2^*) and the degree of association between latent factors (φ), all of which were standardized, were taken into account. From a general perspective, absolute fit and incremental fit indices were contemplated.

The absolute fit indices used were: chi-square (χ^2^), chi-square/degrees of freedom (χ^2^/df), goodness-of-fit index (GFI), adjusted goodness-of-fit index (AGFI), root mean square error of approximation (RMSEA) and standarized root mean square residual (SRMR). χ^2 ^is highly sensitive to sample size [24], for which use was also made of χ^2^/df, which indicates a good fit with a value < 5 or, more strictly, < 3 [20,21,24,25]. GFI measures explained variance and presents the same limitation as χ^2^, while AGFI corrects this limitation depending on the degrees of freedom and number of variables. Both are considered acceptable ≥ 0.9 [26-29]. RMSEA is a measurement of the error of approximation to the population and is considered acceptable < 0.08 [30], although values of < 0.06 [28] and < 0.05 [24] have also been proposed. Generally speaking, values < 0.05 are good, while those close to 0.08 are reasonable and values > 0.1 are unacceptable [31]. SRMR is the standardized difference between the observed and the predicted covariance, indicating a good fit for values < 0.08 [21].

The incremental fit indices used were: normed fit index (NFI), non-normed fit index (NNFI), incremental fit index (IFI) and comparative fit index (CFI). NFI measures the proportional reduction in the adjustment function when going from null to the proposed model; it does not take into account the parsimony of the model and is considered acceptable > 0.9 [32,33]. NNFI considers the degree of freedom of the proposed model and of the independence model and ≥0.9 is recommended [26], although > 0.9 [33] and ≥0.95 [34] have been proposed. IFI also introduces a factor of scale, with values > 0.9 being acceptable [35]. CFI measures improvement in the measurement of non-centrality, also taking into account the parsimony of the model, and indicates good fit ≥0.9 [26], although > 0.9 [30] and ≥0.95 [34] have also been proposed.

Criterial validity was estimated using ROC curve analysis over n_T_. The area under this curve was taken as a representation of the discriminatory capacity of the 'overload', 'lack of development' and 'neglect' dimensions (BCSQ-12) to differentiate between 'cases' and 'non-cases' of 'exhaustion', 'cynicism' and 'lack of efficacy' (MBI-GS), respectively. 'Case'/'non-case' status was established in the criterion dimensions taking as the cut-off the 75 percentile of the standard yardstick for the general Spanish population, corresponding to high or very high scores ('exhaustion'≥2.90; 'cynicism'≥2.26 and 'efficacy'≤3.83) [16]. The χ^2 ^test was used to contrast the area under the ROC curve against the hypothesis of random behaviour. Cut-off points were chosen for the BCSQ-12 dimensions at scores that optimized the sensitivity-specificity ratio, marking the difference between 'exposed' and 'non-exposed' in each of the conditions.

Accuracy was also calculated by means of negative predictive values, overall misclassification rate, positive likelihood ratio tests (coefficient between sensitivity and 1-specificity) and negative likelihood ratio tests (coefficient between 1-sensitivity and specificity). Likelihood ratio tests between 0.5-2 are regarded as poor; between 2-5 or 0.2-0.5 as good; 5-10 or 0.1-0.2 as very good, and > 10 or < 0.1 as excellent [36]. The size of the effect was estimated by using multivariate logistic regression (LR) models by means of the calculation of adjusted Odds ratios (OR), controlling the variables of age, sex, stable relationship, level of education, occupation type, years of service and duration and type of work contract, described in the preceding section. The statistical significance of the effect was estimated by the Wald test and the goodness of fit of models by means of the Hosmer-Lemeshow (H-L) χ^2 ^test. Confidence intervals at 95% (CI 95%) were calculated in all measures of accuracy and effect.

The distribution of items and factors were described by means of the statistical concepts of mean, standard deviation, median, 25-75 percentiles, minimum-maximum scores, asymmetry and kurtosis. Internal consistency was assessed by means of the item-rest correlation, Cronbach's α and according to changes in α through the elimination of each individual item. Contrasts were made depending on sex and occupation using the Mann-Whitney and Kruskal-Wallis tests, given the non-parametric distribution of the dimensions on these groups.

The level of significance adopted in the tests was p < 0.05, and p < 0.017 for multiple comparisons owing to the Bonferroni correction. Data analysis was carried out using the SPSS-15, AMOS-7 and Epidat 3.1 software packages.

## Results

### Characteristics of the study participants

A response rate (RR) of 25.81% was obtained, with 'TRS' (RR = 20.04%) being less participative than 'ASP' (RR = 33.24%) and 'TRA' (RR = 35.76%) (p < 0.001). Table 1 shows the socio-demographic and occupational characteristics of the participants. No significant differences were found between the sub-samples in any of them.

**Table 1:** Characteristics of the study participants

variables	total sample n_T _= 826	sub-sample 1 n_1 _= 413	sub-sample 2 n_2 _= 413	p
**Age**				0.242
Md (SD)	40.26 (9.52)	40.64 (9.59)	39.87 (9,46)	
**Sex**				0.362
male	366 (44.31)	176 (42.62)	190 (46.00)	
**Stable Relationship**				0.999
yes	647 (78.33)	324 (78.45)	323 (78.21)	
**Education**				0.667
secondary	119 (14.41)	64 (15.50)	55 (13.32)	
university	423 (51.21)	208 (50.36)	215 (52.06)	
doctorate	284 (34.38)	141 (34.14)	143 (34.62)	
**Occupation**				0.988
TRS	372 (45.04)	185 (44.79)	187 (45.28)	
ASP	351 (42.49)	176 (42.62)	175 (42.37)	
TRA	103 (12.47)	52 (12.59)	51 (12.35)	
**Length of service**				0.210
< 4 years	184 (22.28)	85 (20.58)	99 (23.97)	
4-16 years	353 (42.74)	172 (41.65)	181 (43.83)	
> 16 years	289 (34.99)	156 (37.77)	133 (32.20)	
**Contract duration**				0.775
permanent	503 (60.90)	254 (61.50)	249 (60.29)	
**Contract type**				0.718
full-time	750 (90.80)	377 (91.28)	373 (90.31)	
**Sick leave**				0.201
yes	256 (30.99)	119 (28.81)	137 (33.17)	

The figures represent frequencies, percentages (in brackets) and the p value associated with an χ^2 ^contrast between sub-sample 1 and sub-sample 2 except for the age variable where the figures represent means, standard deviations and the p value associated with a t contrast.

### Factorial Validity

#### Exploratory Factor Analysis (EFA) over n_1_

All the items presented values of r > 0.30 in the correlations matrix, with 89.39% of them being significant. 83.33% of the MSA were > 0.80 and absolute anti-image values approached 0. The KMO was = 0.83, the matrix determining factor = 0.001 and Bartlett's test p < 0.001. Consequently, the data distribution enabled EFA to be performed legitimately. This analysis provided an unforced solution for three factors. The first ('neglect') explained 37.53% of the variance (eigenvalue = 4.50); the second ('lack of development') explained 20.13% (eigenvalue = 2.41); and the third ('overload') explained 16.12% (eigenvalue = 1.94). The scree test allowed the solution to be accepted as adequate. In total, 73.78% of the variance was explained. Table 2 shows the rotated factor solution and h^2 ^values.

**Table 2:** Exploratory Factor Analysis - weightings and communalities

	Factor			
		
Items	1	2	3	h^2^
3. When things at work don't turn out as well as they should, I stop trying	**0.72**	0.13	0.07	0.54
6. I give up in response to difficulties in my work	**0.85**	0.15	0.14	0.76
9. I give up in the face of any difficulties in my work tasks	**0.73**	0.17	0.14	0.58
12. When the effort I invest in work is not enough, I give in	**0.82**	0.12	0.09	0.70
	
2. I would like to be doing another job that is more challenging for my abilities	0.02	**0.85**	0.05	0.73
5. I feel that my work is an obstacle to the development of my abilities	0.29	**0.68**	0.22	0.62
8. I would like to be doing another job where I can better develop my talents	0.12	**0.92**	0.04	0.86
11. My work doesn't offer me opportunities to develop my abilities	0.22	**0.72**	0.02	0.58
	
1. I think the dedication I invest in my work is more than what I should for my health	0.07	0.13	**0.80**	0.67
4. I neglect my personal life when I pursue important achievements in my work	0.09	0.02	**0.82**	0.67
7. I risk my health when I pursue good results in my work	0.06	0.01	**0.77**	0.60
10. I overlook my own needs to fulfil work demands	0.20	0.11	**0.68**	0.52

Extraction method: Maximum Likelihood with Varimax orthogonal rotation on sub-sample 1. h^2 ^= communalities. Bold = belonging factor

#### Confirmatory Factor Analysis (CFA) over n_2_

**Figure 1:**
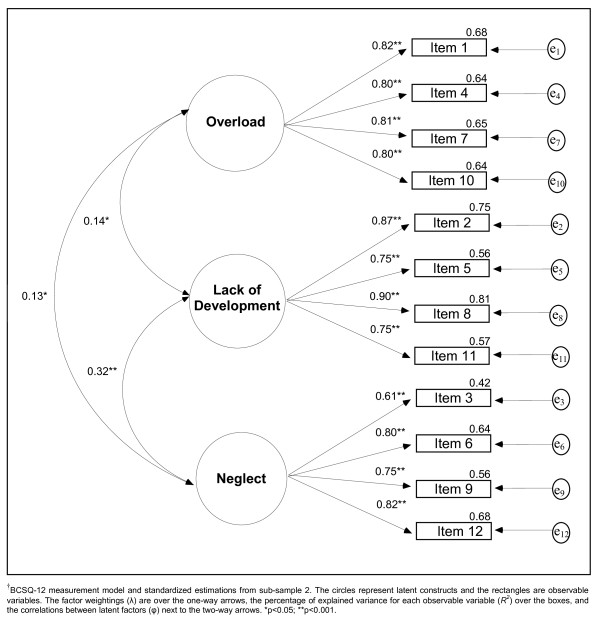
**Analytical perspective of Confirmatory Factor Analysis^†^**.

Mardia's coefficient was = 66.77 (p < 0.001), which made it possible to use the ML estimation method in conditions of distance from the assumption of multivariate normality. Figure 1 shows the results of CFA from an analytical perspective. The fit indices for this model were: χ^2 ^= 149.61 (gl = 51; p < 0.001), χ^2^/gl = 2.93, GFI = 0.941, AGFI = 0.911, RMSEA = 0.068 (90% CI = 0.055-0.080), SRMR = 0.059, NFI = 0.943, NNFI = 0.951, IFI = 0.962 and CFI = 0.962. The entry into the model of those correlations between the error terms with modification indices that showed significant reductions in the value of χ^2 ^[e_4_-e_5 _(r = 0.13; p = 0.015), e_4_-e_10 _(r = 0.19; p = 0.009), e_5_-e_6 _(r = 0.18; p = 0.002), e_5_-e_11 _(r = 0.20; p < 0.001) y e_6_-e_11 _(r = 0.15; p = 0.014)], gave the following indices: χ^2 ^= 112.04 (gl = 46; p < 0.001), χ^2^/gl = 2.44, GFI = 0.958, AGFI = 0.929, RMSEA = 0.059 (90% CI = 0.045-0.073), SRMR = 0.057, NFI = 0.958, NNFI = 0.963, IFI = 0.975 and CFI = 0.974.

### Criterial validity

When predicting 'exhaustion', the area under the ROC curve for 'overload' was = 0.75, this was = 0.80 for 'lack of development' relative to 'cynicism' and = 0.74 for 'neglect' relative to 'inefficacy' (p < 0.001). Table 3 shows the accuracy of cut-off points that optimized the sensitivity-specificity ratio ['overload'≥3.38 (se = 75.89; sp = 62.35); 'lack of development'≥3.63 (se = 70.71; sp = 70.57); 'neglect'≥2.63 (se = 71.19; sp = 67.03)].

**Table 3:** Exactness of BCSQ-12 according to MBI-GS

	criterion: 'exhaustion' (cut-off point 'overload'≥3.38)		criterion: 'cynicism' (cut-off point 'L.development'≥3.63)		criterion: 'inefficacy' (cut-off point 'neglect'≥2.63)	
	
	index	95% IC	index	95% IC	index	95% IC
Sensitivity *	75.89	70.07 - 81.72	70.71	65.21 - 76.22	71.19	64.23 - 78.14
Specificity *	62.35	58.40 - 66.30	70.57	66.66 - 74.48	67.03	63.33 - 70.72
PPV^a ^*	42.82	37.83 - 47.81	55.15	49.87 - 60.44	37.06	31.78 - 42.34
NPV^b ^*	87.44	84.19 - 90.69	82.48	78.93 - 86.06	89.51	86.68 - 92.33
OMR^c ^*	33.98	30.69 - 37.27	29.38	26.22 - 32.55	32.08	28,84 - 35.33
PLR^d^	2.02	1.78 - 2.29	2.40	2.07 - 2.79	2.16	1.87 - 2.49
NLR^e^	0.39	0.30 - 0.49	0.42	0.34 - 0.50	0.43	0.34 - 0.55
OR^f^	5.25^g^	3.62 - 7.60	6.77^h^	4.79 - 9.57	5.21^i^	3.57 - 7.60

*values given as percentages. a = Positive predictive value. b = Negative predictive value. c = Overal misclassification rate. d = Positive likelihood ratio. e = Negative likelihood ratio. f = Adjusted Odds Ratio by means of multivariate logistic regression models controlling age, sex, stable relationship, education, occupation, length of service, contract duration and contract type. g = Wald p < 0.001; H-L p = 0.451. h = Wald p < 0.001; H-L p = 0.093. i = Wald p < 0.001; H-L p = 0.216. Values obtained from the total sample (n_T_).

### Descriptives, internal consistency and contrasts

25.06% of participants in the total sample presented high or very high scores in only one of the MBI-GS dimensions; 16.46% did so in two of them; and 8.11% in all three. Table 4 shows the descriptives for the BCSQ-12 items, while Table 5 shows those corresponding to the BCSQ-12 and MBI-GS dimensions, as well as contrast with regard to sex and occupation. The results of the internal consistency analysis showed that removal of items separately caused the α value to decrease in all cases. No differences were found with regard to sex, but there were differences depending on occupation. Teaching or research staff (TRS) showed higher levels of 'exhaustion' than administration or service personnel (ASP), TRS and trainees (TRA) presented higher levels of 'overload', ASP showed higher levels of 'lack of development' (p < 0.001). TRA showed lower levels of 'neglect' than ASP (p = 0.004).

**Table 4:** Descriptive statistics for BCSQ-12 items

items	Mn	SD	Q_1_	Mdn	Q_3_	min	max	asym^a^	kurt^b^	Item-rest
1. I think the dedication I invest in my work is more than what I should for my health	3.83	1.66	3.00	4.00	5.00	1.00	7.00	0.09	-0.80	0.75
4. I neglect my personal life when I pursue important achievements in my work	3.10	1.71	2.00	3.00	4.00	1.00	7.00	0.57	-0.56	0.75
7. I risk my health when I pursue good results in my work	3.43	1.69	2.00	3.00	5.00	1.00	7.00	0.33	-0.73	0.74
10. I overlook my own needs to fulfil work demands	3.53	1.63	2.00	3.00	5.00	1.00	7.00	0.21	-0.73	0.69

2. I would like to be doing another job that is more challenging for my abilities	3.42	1.86	2.00	3.00	5.00	1.00	7.00	0.31	-0.90	0.77
5. I feel that my work is an obstacle to the development of my abilities	3.08	1.64	2.00	3.00	4.00	1.00	7.00	0.61	-0.29	0.72
8. I would like to be doing another job where I can better develop my talents	3.68	1.86	4.00	4.00	5.00	1.00	7.00	0.14	-1.01	0.82
11. My work doesn't offer me opportunities to develop my abilities	3.53	1.86	2.00	3.00	5.00	1.00	7.00	0.30	-0.96	0.73

3. When things at work don't turn out as well as they should, I stop trying	2.46	1.26	1.00	2.00	3.00	1.00	7.00	0.92	1.08	0.61
6. I give up in response to difficulties in my work	2.36	1.24	1.00	2.00	3.00	1.00	7.00	0.88	0.90	0.74
9. I give up in the face of any difficulties in my work tasks	2.12	1.09	1.00	2.00	3.00	1.00	7.00	1.05	1.84	0.68
12. When the effort I invest in work is not enough, I give in	2.48	1.20	1.00	3.00	3.00	1.00	7.00	0.69	0.64	0.74

Mn = mean. SD = standard deviation. Mdn = median. Q_1 _= percentile-25. Q_3 _= percentile-75. min = minimum score. max = maximum score. asym = asymmetry. kurt = kurtosis. Item-rest = correlation coefficient item-rest (r between each item and the remaining items belonging to the same factor). a = typical asymmetry error = 0.08 for all items. b = typical kurtosis error = 0.17 for all items. Values obtained from the total sample (n_T _= 826).

**Table 5:** Descriptive statistics, Cronbach's α and contrasts with regard to sex and occupation for the BCSQ-12 and MBI-GS dimensions

		BCSQ-12			MBI-GS		
		
	(n)	Overload	L. Development	Neglect	Exhaustion	Cynicism	Efficacy
**Total**	826						
*Mn*		3.47	3.43	2.35	2.24	2.01	4.47
*SD*		1.42	1.57	1.00	1.42	1.57	0.97
*Mdn*		3.25	3.25	2.25	2.00	1.50	4.58
*Q_1_*		2.50	2.25	1.50	1.20	0.75	3.83
*Q_3_*		4.50	4.50	3.00	3.20	3.00	5.17
*min*		1.00	1.00	1.00	0.00	0.00	0.00
*max*		7.00	7.00	6.25	6.00	6.00	6.00
*asym^a^*		0.34	0.28	0.48	0.71	0.78	-0.72
*kurt^b^*		-0.50	-0.62	0.06	-0.14	-0.23	0.71
*α*		0.87	0.89	0.85	0.91	0.92	0.82

**Male**	366						
*Mdn*		3.25	3.50	2.25	1.80	1.75	4.50
*Q_1_*		2.50	2.25	1.50	1.00	1.00	3.83
*Q_3_*		4.50	4.62	3.00	3.00	3.00	5.17
*α*		0.86	0.88	0.86	0.91	0.91	0.81
**Female**	460						
*Mdn*		3.25	3.25	2.50	2.00	1.50	4.67
*Q_1_*		2.50	2.25	1.50	1.00	1.00	3.83
*Q_3_*		4.50	4.25	3.00	3.20	2.94	5.17
*α*		0.88	0.89	0.84	0.92	0.92	0.83

*p*^c^		0.502	0.082	0.480	0.194	0.108	0.124

**TRS**	372						
*Mdn*		3.75	3.00	2.25	2.00	1.50	4.50
*Q_1_*		3.00	1.75	1.50	1.40	0.75	3.83
*Q_3_*		5.00	4.00	3.00	3.60	3.00	5.00
*α*		0.87	0.86	0.84	0.92	0.92	0.82
**ASP**	351						
*Mdn*		3.00	4.00	2.50	1.80	1.75	4.67
*Q_1_*		2.25	3.00	1.50	1.00	1.00	4.00
*Q_3_*		3.50	5.00	3.00	2.80	3.00	5.17
*α*		0.85	0.90	0.86	0.90	0.91	0.82
**TRA**	103						
*Mdn*		3.50	3.00	2.00	2.00	1.50	4.50
*Q_1_*		2.50	1.75	1.25	1.00	0.75	3.67
*Q_3_*		5.25	4.00	2.75	3.40	2.75	5.50
*α*		0.87	0.91	0.86	0.93	0.94	0.85

*p*^d^		**< 0.001**	**< 0.001**	**0.016**	**0.006**	0.305	0.155

TRS *vs *ASP		**< 0.001**	**< 0.001**	0.322	**0.001**	0.123	0.056
TRS *vs *TRA		0.456	0.622	0.023	0.466	0.786	0.344
ASP *vs *TRA		**< 0.001**	**< 0.001**	**0.004**	0.202	0.501	0.863

Mn = mean. SD = standard deviation. Mdn = median. Q_1 _= percentile-25. Q_3 _= percentile-75. min = minimum score. max = maximum score. asym = asymmetry. kurt = kurtosis. a = typical asymmetry error = 0.08. b = typical kurtosis error = 0.17. c = Mann-Whitney contrast. d = Kruskal-Wallis contrast.

## Discussion

The BCSQ-12 has been proposed as a definition of burnout that could cover common ground between the typological and standard approaches [1,2,4,7,8]. Its factor and criterial validity had not been tested until now. By using a multi-occupational sample of university employees, EFA and CFA were performed on different sub-samples, a ROC curve analysis was carried out with the MBI-GS as a standard criterion and a contrast of hypotheses was made for both models with respect to sex and occupation.

The prevalence values obtained for the study sample according to the classical dimensions were high, although within the expected range. The structure of the BCSQ-12 behaved consistently throughout the factor analyses. All the items loaded perfectly on the factors following the original design, and they were all well explained. Internal consistency was very good in all cases and all items contibuted to its increase. The restrictions imposed by the model were well fitted to all the data, from both an absolute and incremental perspective. The discriminatory capacity of the classifier and the accuracy associated with the proposed cut-off points were good. The sensitivity and specificity shown by the dimensions of the BCSQ-12 when predicting those of the MBI-GS do not show the values that we normally expect to obtain from an ideal classifier, however, they are seen to be moderately high and all significant, far from those of random behaviour. Although the likelihood of being a 'non-case' among unexposed subjects offered an excellent score that of being a 'case' among exposed subjects offered a more limited score, which made the misclassification increase in this sense. Nevertheless, the likelihood of being a 'case' among exposed subjects was much greater than those who were not exposed, the likelihood of attaining the status of 'exposed' was greater among the 'cases' and the likelihood of attaining the status of 'unexposed' was greater among 'non-cases'. No significant differences were found with regard to sex, but there were differences depending on occupation. 'TRS' showed higher levels of 'exhaustion' than 'ASP'. 'TRS' and 'TRA' presented higher levels of 'overload' and ASP showed higher levels of 'lack of development'. 'TRA' showed lower levels of 'neglect' than 'ASP'.

As limitations to the study, we should mention that the scores for variables considered were self-reported and therefore may have been weakened by the effects of socially desirable responses. The utilization of a sample obtained from a sole organization may have limited the external validity of the obtained results. Still, this is a broad and multi-occupational sample made up of workers with very diverse jobs, which reinforces the possibility of generalization. Certainly, the RRs obtained with regard to occupation were different and could have introduced a possible selection bias that may have affected the representative nature of the sample. However, we would also mention that this does not produce an important reduction in the statistical power for comparing the groups. We found that teaching and research staff were significantly less participative than administration and service personnel and trainees. Nonetheless, all the response rate values obtained from these groups, although low, fell within the range that could be expected when using this data collection procedure [10,11]. Our opinion is that this pattern of response could be due to differences in the type of burnout mostly present in each occupational category, which follows the line put forward by Montero-Marín et al. [4] and is in agreement with the results obtained in this study concerning the differences between groups. The fact that teaching and research staff show a greater tendency to suffer from overload may influence their being less participative, owing to the little time they have and their strong focus on accomplishing their own goals. Administration and service personnel, showing a greater tendency to experience lack of development, would appear to be more participative perhaps as this allows them a momentary break from the monotony of their work. The trainees, showing outstandingly low levels of neglect, appear to be a participative group, most likely owing to the nature of their jobs and to their scarce exposure in time to the rigidity of the organizational structure of the institution, which would leave them feeling less worn out. Consequently, the different response rates obtained depending on occupational categories could be explained in relation to the differences between the burnout types encountered. This point gains in importance if we are to obtain representative samples for the calculation of prevalence values for burnout syndrome depending on the different occupational strata [5]. Therefore, this will have to be taken into account when recruiting participants in future research projects. Finally, the criterion was established from a psychometric level, given the lack of consensus in the contemporary scene from a clinical perspective. As strengths of the study, we would underscore the quality of the data, which was controlled by eliminating the possible errors from the transcription process by means of purpose-designed software. Likewise, the obtention of convergent results between exploratory and confirmatory analyses, carried out on different sub-samples, increases the confidence of our results.

According to social exchange theory, the establishment of reciprocal relations is essential for the health and well-being of individuals. Perception of the lack of reciprocity in a work environment plays a fundamental role in the development of burnout syndrome and increases the risk of individuals suffering from emotional disorders [37-39]. This is due to the imbalance between effort and gratification being an important source of stress [40]. The manifestation of burnout through different clinical subtypes corresponds to coping with feelings of frustration produced through differing levels of commitment [3-8].

Individuals suffering from "frenetic" burnout experience the feeling of 'overload' when they try to maximize their rewards by taking on a volume and pace of work that become excessive [3-8]. This feeling constitutes a classic aetiological factor of burnout [41-43], which was observed to be associated with 'exhaustion' in our study. According to Karasek's model, high demands and low autonomy in the workplace increase exhaustion levels and thus the likelihood of developing the syndrome, particularly in workers with poor time management skills and a low level of resources [44-46]. The "frenetic" subtype offers a profile of active coping that could benefit from interventions directed at reducing activation, for the purpose of removing accumulated tension and preventing exhaustion; improvement in time management to make room for the total satisfaction of personal needs; and development of self-assertion in order to place limits on the acceptance of responsibilities.

The "underchallenged" subtype balances rewards by carrying out tasks in a superficial manner, leading to feelings of meaninglessness and lack of personal development in the workplace [3-8]. This has an influence on the negative assessment of work conditions [47], constitutes a risk factor for burnout [48,49] and has been associated with boredom, indifference and a mechanical performance [8]. It has been associated with 'cynicism' in our study. From a non-linear perspective, Karasek's model explains the origin of feeling of frustration as the absence of challenges resulting from monotony owing to low demands in the workplace [50]. The "underchallenged" subtype, situated between active and passive coping modes although closer to the latter, may benefit from interventions that encourage interest, satisfaction and personal development through training of conscious attention towards tasks and through the establishment of challenging and significant targets.

The "worn-out" subtype optimizes rewards by reducing efforts through 'neglect' of responsibilities and chooses this as a consequence of the defencelessness learned in the individual's experience with the organization [3-8]. This 'neglect' is the opposite of commitment [7,51] and is seen in our study to be associated with the perception of 'lack of efficacy' in the carrying out of tasks. According to Karasek's model, experiences of lack of control play an important part in the health of workers and reduce their productivity [44,52], leading to a breaking of an individual's commitment through the erosion they cause in expectations of self-efficacy, given the modulating role these play in the maintenance of behaviours [53,54]. The "worn-out" subtype presents a profile of passive coping that could benefit from interventions directed at treatment for despair and increased confidence through the regaining of control and the perception of self-efficacy.

A definition of the syndrome that is able to discriminate the type of experienced burnout by means of the identification of clinical profiles according to a three-dimensional definition, such as that presented in the BCSQ-12, offers understanding into the type of dysfunctional attitudes associated with each case, favouring the development of more specific interventions within a conceptual framework according to the classical perspective. From our point of view, this is due to the fact that the model provided by the BCSQ-12 extends the standard definition of burnout, allowing greater differentiation to be made using clinical subtypes; but at the cost of becoming a little distanced from the core of the syndrome as it has been considered using the classical model. Extra validity will be given to the proposed model through the clinical benefits that this new definition may produce by means of the design of new and more specific interventions for the syndrome.

Our study shows how the BCSQ-12 went further than the standard MBI-GS in characterizing work-related discomfort experienced with regard to occupation. Taking into account the series of inconsistencies presented by the classic standard [55,56], the BCSQ-12 may provide a more solid definition of the syndrome at a structural level. The therapeutic interventions derived from the standard model has not produced very promising results to date [57], perhaps because not enough attention has been given to the matter of the type of dissatisfaction and burnout experienced. Generally speaking, the evidence shows that levels of satisfaction in the workplace have a decisive influece on the health of workers [58]. Future research will need to clarify whether this new perspective will be able to produce more effective interventions for burnout and for the improvement of workers' health status.

## Conclusions

Our results provide evidence in favour of the criterial and construct validity of the brief typological definition of burnout established in BCSQ-12. This questionnaire can be a very useful instrument for future evaluation and also for designing interventions, as it provides an approach to the syndrome focusing on the identification of the type of dissatisfaction and discomfort experienced.

## Competing interests

The authors declare that they have no competing interests.

## Authors' contributions

JMM, JGC, PS, RA and MG conceived the study design. JMM and JGC collected the data, JMM, PS, JGC and RA conducted the statistical analysis, REDIAPP has given scientific and statistical support over the research study and all authors contributed to the interpretation of the results, the drafting of the manuscript, and the approval of the final manuscript.

## Supplementary Material

Additional file 1 **Appendix 1. "Burnout Clinical Subtype Questionnaire" (BCSQ-12), Spanish version**. The BCSQ-12 in its English version is presented and scoring explained to facilitate the use by the readers. Appendix 2. "Burnout Clinical Subtype Questionnaire" (BCSQ-12), English version. The BCSQ-12 in its Spanish version is presented and scoring explained to facilitate the use by the readers. Click here for file
