# Supplementary material for: Towards a brief definition of burnout syndrome by subtypes: Development of the "Burnout Clinical Subtypes Questionnaire" (BCSQ-12)
Source: Health Qual Life Outcomes. 2011 Sep 20;9:74. doi: 10.1186/1477-7525-9-74 (PMC3196899; doi:10.1186/1477-7525-9-74)
Supplement: Additional file 1 — Appendix 1. "Burnout Clinical Subtype Questionnaire" (BCSQ-12), Spanish version. The BCSQ-12 in its English version is presented and scoring explained to facilitate the use by the readers. Appendix 2. "Burnout Clinical Subtype Questionnaire" (BCSQ-12), English version. The BCSQ-12 in its Spanish version is presented and scoring explained to facilitate the use by the readers. [file 1477-7525-9-74-S1.DOC]

**APPENDIX 1**

**“Burnout Clinical Subtype Questionnaire” (BCSQ-12)**

The following is a series of statements indicating experiences that may occur at work. Read each statement carefully and mark with an X the option that best represents how you feel, what you do and what you think about your work. There are no right or wrong answers. Please **DO NOT LEAVE ANY STATEMENT UNANSWERED.**

|  | **Totally disagree** | **Strongly disagree** | **Disagree** | **Unsure** | **Agree** | **Strongly agree** | **Totally**  **agree** |
| --- | --- | --- | --- | --- | --- | --- | --- |
| 1. I think the dedication I invest in my work is more than what I should for my health | O | O | O | O | O | O | O |
| 2. I would like to be doing another job that is more challenging for my abilities | O | O | O | O | O | O | O |
| 3. When things at work don’t turn out as well as they should, I stop trying | O | O | O | O | O | O | O |
| 4. I neglect my personal life when I pursue important achievements in my work | O | O | O | O | O | O | O |
| 5. I feel that my work is an obstacle to the development of my abilities | O | O | O | O | O | O | O |
| 6. I give up in response to difficulties in my work | O | O | O | O | O | O | O |
| 7. I risk my health when I pursue good results in my work | O | O | O | O | O | O | O |
| 8. I would like to be doing another job where I can better develop my talents | O | O | O | O | O | O | O |
| 9. I give up in the face of any difficulties in my work tasks | O | O | O | O | O | O | O |
| 10. I overlook my own needs to fulfil work demands | O | O | O | O | O | O | O |
| 11. My work doesn’t offer me opportunities to develop my abilities | O | O | O | O | O | O | O |
| 12. When the effort I invest in work is not enough, I give in | O | O | O | O | O | O | O |

**Correction algorithm:**

The ‘overload’ dimension is made up of items 1, 4, 7, 10.

The ‘lack of development’ dimension is made up of items 2, 5, 8, 11.

The ‘neglect’ dimension is made up of items 3, 6, 9, 12.

The answers are scored between 1 (totally disagree) and 7 (totally agree).

**APPENDIX 2**

**“Cuestionario de Subtipos Clínicos de Burnout” (BCSQ-12)**

A continuación se presentan una serie de enunciados que indican vivencias que puede experimentar en el trabajo. Lea cada frase con atención y señale con una X la opción que mejor represente cómo se siente, lo que hace o lo que piensa respecto a su actividad laboral. No existen respuestas correctas o incorrectas. Por favor, **NO DEJE NINGUNA RESPUESTA SIN CONTESTAR**.

|  | **Totalmente en desacuerdo** | **Muy en desacuerdo** | **En desacuerdo** | **Indeciso** | **De acuerdo** | **Muy de acuerdo** | **Totalmente de**  **acuerdo** |
| --- | --- | --- | --- | --- | --- | --- | --- |
| 1. Creo que invierto más de lo saludable en mi dedicación al trabajo | O | O | O | O | O | O | O |
| 2. Me gustaría dedicarme a otro trabajo que planteara mayores desafíos a mi capacidad | O | O | O | O | O | O | O |
| 3. Cuando las cosas del trabajo no salen del todo bien dejo de esforzarme | O | O | O | O | O | O | O |
| 4. Descuido mi vida personal al perseguir grandes objetivos en el trabajo | O | O | O | O | O | O | O |
| 5. Siento que mi actividad laboral es un freno para el desarrollo de mis capacidades | O | O | O | O | O | O | O |
| 6. Me rindo como respuesta a las dificultades en el trabajo | O | O | O | O | O | O | O |
| 7. Arriesgo mi salud en la persecución de buenos resultados en el trabajo | O | O | O | O | O | O | O |
| 8. Me gustaría desempeñar otro trabajo en el que pudiera desarrollar mejor mi talento | O | O | O | O | O | O | O |
| 9. Abandono ante cualquier dificultad en las tareas de mi trabajo | O | O | O | O | O | O | O |
| 10. Ignoro mis propias necesidades por cumplir con las demandas del trabajo | O | O | O | O | O | O | O |
| 11. Mi trabajo no me ofrece oportunidades para el desarrollo de mis aptitudes | O | O | O | O | O | O | O |
| 12. Cuando el esfuerzo invertido en el trabajo no es suficiente, me doy por vencido | O | O | O | O | O | O | O |

**Algoritmo de Corrección:**

La dimensión ‘sobrecarga’ está constituida por los ítems nº: 1, 4, 7, 10.

La dimensión ‘falta de desarrollo’ está constituida por los ítems nº: 2, 5, 8, 11.

La dimensión ‘abandono’ está constituida por los ítems nº: 3, 6, 9, 12.

Las respuestas se valoran de 1 (totalmente en desacuerdo) a 7 (totalmente de acuerdo).
